# Advance directives for future dementia can be modified by a brief video presentation on dementia care: An experimental study

**DOI:** 10.1371/journal.pone.0197229

**Published:** 2018-05-24

**Authors:** Theresia Volhard, Frank Jessen, Luca Kleineidam, Steffen Wolfsgruber, Dirk Lanzerath, Michael Wagner, Wolfgang Maier

**Affiliations:** 1 German Reference Centre for Ethics in the Life Sciences (DRZE), Bonn, Germany; 2 Department of Psychiatry and Psychotherapy, University of Cologne, Cologne, Germany; 3 German Center for Neurodegenerative Diseases (DZNE), Bonn, Germany; 4 Department of Neurodegenerative Diseases and Geriatric Psychiatry, University of Bonn, Bonn, Germany; Istituto Di Ricerche Farmacologiche Mario Negri, ITALY

## Abstract

**Objectives:**

To investigate whether life-sustaining measures in medical emergency situations are less accepted for an anticipated own future of living with dementia, and to test whether a resource-oriented, in contrast to a deficit-oriented video about the same demented person, would increase the acceptance of such life-saving measures.

**Design:**

Experimental study conducted between September 2012 and February 2013.

**Setting:**

Community dwelling female volunteers living in the region of Bonn, Germany.

**Participants:**

278 women aged 19 to 89 (mean age 53.4 years).

**Intervention:**

Presentation of a video on dementia care focusing either on the deficits of a demented woman (negative framing), or focusing on the remaining resources (positive framing) of the same patient.

**Main outcome measures:**

Approval of life-sustaining treatments in five critical medical scenarios under the assumption of having comorbid dementia, before and after the presentation of the brief videos on care.

**Results:**

At baseline, the acceptance of life-sustaining measures in critical medical situations was significantly lower in subjects anticipating their own future life with dementia. Participants watching the resource-oriented film on living with dementia had significantly higher post-film acceptance rates compared to those watching the deficit-oriented negatively framed film. This effect particularly emerges if brief and efficient life-saving interventions with a high likelihood of physical recovery are available (eg, antibiotic treatment for pneumonia).

**Conclusions:**

Anticipated decisions regarding life-sustaining measures are negatively influenced by the subjective imagination of living with dementia, which might be shaped by common, unquestioned stereotypes. This bias can be reduced by providing audio-visual information on living with dementia which is not only centred around cognitive and functional losses but also focuses on remaining resources and the apparent quality of life. This is particularly true if the medical threat can be treated efficiently. These findings have implications for the practice of formulating, revising, and supporting advance directives.

## Introduction

Persons suffering from dementia experience progressive limitations in their cognitive and decision-making capacity. Inevitably, their ability to make autonomous and informed decisions will be lost [1–3].

The Convention of the United Nations on the rights of persons with disabilities very clearly allocates the priority to the self-determination autonomy of the person—also in case of severely disabling disease and illness [4]. A valid process of advance decision-making for states of limited cognitive capacity and particularly for severe dementia needs to fulfil criteria of a well-informed frame of support for decision-making to avoid a later substituting decision-making. The protection of the autonomy of a person in a state of incompetence for medical decisions is a moral demand—particularly as medical decision-making is traditionally based on the medical judgement of a physician and/or the wishes of relatives who consider themselves to be the guardian of the patient [5]. The current medical practice at many places often does not meet the wishes of patients [6]. Stimulated by the UN convention, the priority is increasingly allocated to the autonomy of a person, even if she/he is in an incompetent state.

Advance directives are the most widely accepted tool for safeguarding a—still competent—person’s anticipated medical decisions in expectation of suffering dementia later in life [7]. Preferences expressed in such manner are considered to reflect enduring personal values and critical interests [8]. An advance directive is used mainly to refuse medical interventions [9]. A refusal of treatment expressed in an unrevoked advance directive is generally viewed as the patient’s autonomous wish which is perceived as valid also in the future—if it is not revoked [10]. Even if the person’s advance decision is thought to be irrational from the perspective of medical professionals, and even if the decision leads to an earlier death of the patient, the patient’s autonomy has to be respected. Advance directives are strongly recommended for the case of experiencing dementia [11].

This development in medical ethics creates a serious challenge to the medical system and requires changes of treatment habits. These demands are indicated by a survey in the UK: only a minority of competent adults wants any life-sustaining treatments after a heart attack in case they are living with severe dementia [12].

In practical terms, the priority for patient’s autonomy requires feasible and accepted instruments for self-determination in future critical situations where decisional competence has been lost [13]. In this context, precedent autonomy is a core component of the concept of personal autonomy: "precedent autonomy is the right of a competent individual to make decisions based on the "critical interests" for a later time once competence has been lost" [14]. Critical interests encompass personal dignity, integrity and well-being expressed by the competent person. Precedent autonomy of a competent person takes precedence over preferences in later stages of decisional incompetence. In many legal systems, advance directives are implemented as a binding instrument allowing to refuse treatment.

Advance directives are getting continuously more important, as they are an integrative component of advance care planning. Advance care programmes are internationally promoted as an efficient strategy to improve care for people with dementia [13, 15]. Yet, the effectiveness and acceptability of these programmes are limited [16]. The broader implementation of advance directives is a feasible first step to promote advance care planning.

Although a majority in the general population and among health care professionals supports the rationale of advance directives, the applications on individual real life conditions are often not unequivocal. This lack of certainty is reflected by a recent empirical investigation showing that a substantial minority (about one quarter) of carers and health professionals would disregard an advance refusal of treatment [17]. A main reason for this disregard is the "past-directive versus present interests" dilemma: an executed advance treatment refusal for the case of later decisional incapability is in contrast to the positive emotional status of the demented patient in a medically critical situation [17]. Ethically it is also considered as questionable if an advance directive can be revoked by behavioural expressions after the loss of the decision-making capacity [18].

In this context, several critical issues have to be considered:

1. Subjective and societal values and attitudes, self-concepts and end-of-life views and medical interventions may change during the temporal gap between drafting the advance directive and its application. Thus, epistemically the future may be too opaque for a future binding decision [19].
2. It is difficult to anticipate how it is to live in an cognitively incompetent state with dementia. This projection requires a change of self-evaluation beyond everyday experience. This challenge may be difficult to achieve. Furthermore, an incompetent and ill person may no longer possess the desires and preferences on which his/her advance directives were previously based. The adaptation to new health conditions with changed preferences due to acquired disabilities is a common phenomenon ("hedonic adaption") [20]. Particularly, there is empirical evidence that preferences for life-sustaining treatments are dependent on the context in which they are made, and thus individuals may express different treatment preferences when they are healthy than when they are ill [21].
3. Persons with dementia may unexpectedly cope with the new, previously unfamiliar condition and may demonstrate well-being, despite the anticipation—in a competent state—not to tolerate the loss of independence, of self-control and of rationality. For example, there is empirical evidence for change of treatment preferences when experiencing serious illness including dementia ("response shift") [22]. Thus it has been argued that the patient´s autonomy is not respected by honouring the desires he/she used to have previously but not so currently in an incompetent state of dementia [23].
4. The societal views of dementia are likely to influence the autonomous person delivering an advance directive. Most prevalent is a "negative", deficit-oriented image of dementia focussing on the insufficiencies, decay and deteriorated quality of life (dementia as debilitating, degrading disease). This attitude produced the stigma on the diagnosis of dementia [24]. The resulting negative image of dementia is oversimplified and fixed, it is persistent since decades: it became a "stereotype". The alternative view is a "positive", resource-oriented image, focusing on positive emotions, interpersonal esteem and subjective well-being. Even so less frequently accepted, this view is more realistic at least for a substantial proportion of people living with dementia in more supportive and favourable surroundings.

Given these uncertainties, erroneous anticipations for a future status of incompetence are possible [17]. This multifacetted constellation was characterised as a "dilemma" [25]. The relevance of this dilemma is increasing: (a) given that methods for pre-clinical diagnosis, at least of Alzheimer’s disease, are already in existence and will probably be a part of clinical practice soon [26], (b) given the development of drugs intervening efficiently in the course of dementia also in later stages of Alzheimer’s disease [27].

Here, we investigate two aspects:

In a first step we test whether attitudes towards anticipated decisions regarding life-sustaining measures in a medical emergency situation differ depending on the anticipated mental state (ie, with anticipated dementia versus without dementia). We hypothesise that in the same medical situations (presented by case vignettes) life-extending measures are more frequently rated in favour of an intervention when absence of dementia is imagined as compared to an imagined condition of dementia (hypothesis 1).
In a second step we investigate whether attitudes towards life-sustaining measures can be modified by presenting different views of living with dementia by video films. Two alternative differently valued perspectives can be taken: one visualising a positive, resource-oriented perspective and another one visualising the stereotype of a negative, deficit-oriented perspective on dementia. We hypothesize that attitudes towards life-saving interventions in case of late-stage dementia are dependent on the transported view on this illness (hypothesis 2).

## Methods

### Participants

For this study a diverse sample of 278 female, cognitively competent healthy participants was recruited, including women across a broad age range (mean = 53.4 years, SD = 17.6), with and without experience with dementia care. We focussed on female participants because women are more often afflicted with dementia than men, partly due to their higher life-expectancy [13].

Participants were recruited through advertisements in newspapers and flyers delivered to public places (nursing homes, counselling centres, vocational schools, cafés, supermarkets, etc.). All subjects received a small compensation of 25 € for their participation in the study and gave written informed consent. All procedures were conducted in research facilities of the German Reference Centre for Ethics in the Life Sciences (DRZE), Bonn, Germany. The study protocol was approved by the Ethical Committee of the University of Bonn (ID 105/12).

### Experimental design and randomization

**Fig 1:**
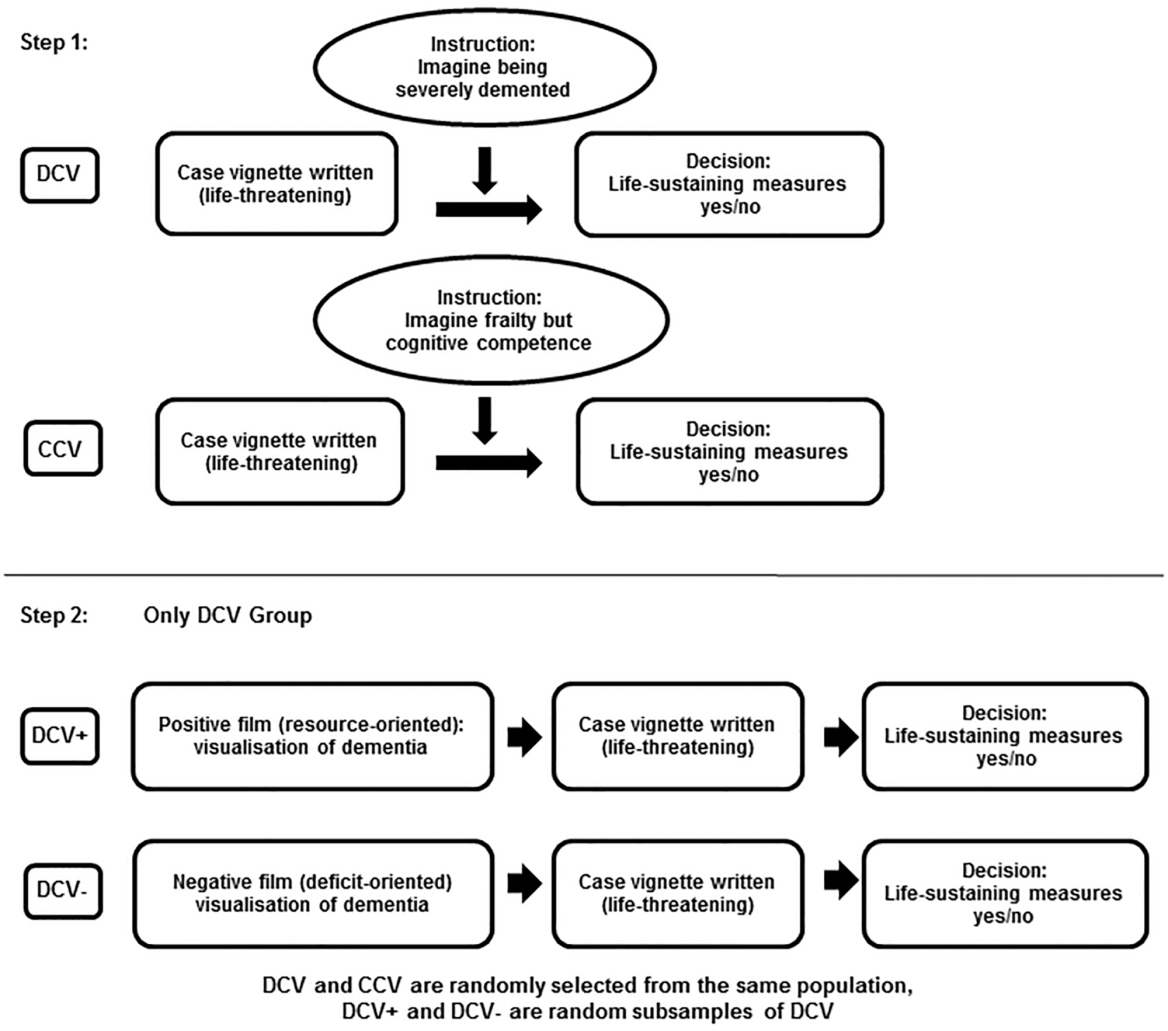
Two-step experimental design. DCV: Dementia Case Vignette; CCV: Control Case Vignette; DCV+: Dementia Case Vignette after seing a resource-oriented film on living with dementia ("positive film"); DCV-: Dementia Case Vignette after seeing a deficit-oriented film on living with dementia ("negative film").

Participants were randomized in the first step (Fig 1) into two groups according to a 2:1 ratio. Both groups were presented with the same series of five case vignettes of life-threatening medical situations with the request to agree or disagree with life-sustaining measures as detailed below. The first group of participants was instructed to imagine being in an advanced dementia state before considering the case vignettes ("DCV"), while the second group (control case vignettes "CCV") received the instruction to imagine being in need for care due to old-age-related physical frailty (ie, requiring help with personal care, household, eating), while being mentally unaffected. Participants exposed to the DCV vignettes (abbreviation: DCV group) received a description of advanced dementia as a condition characterized by severe memory loss (particularly of short-term memory), language impairment and difficulties to recognise even close relatives. The CCV group received a description of living with frailty in old age characterized by total dependency on others including the inability to perform even simple daily activities like eating and personal hygiene routines. All participants were then asked to imagine themselves in such a chronic condition (i.e. being demented or frail, respectively) and to further assume that, in the acute situation depicted in the clinical vignettes, they would not be capable to decide autonomously whether a medical intervention should be undertaken or not (which is the standard imagination invoked for advance directives).

A second step (Fig 1) explores the modifiability of choices taken in step 1 by providing two paradigms of emotionally valued information on living with dementia (negative: deficit-oriented; positive: resource-oriented). For this purpose only the DCV group was randomly split in two subgroups of equal size.

One subgroup (DCV+) watched the brief, resource-oriented film clip on living with dementia, the other subgroup watched the deficit-oriented film clip on living with dementia (see Presentation of videos below). Both groups were then asked again to make decisions regarding life-sustaining measures in the five clinical vignettes with the different life-threatening situation (identical with step 1).

### Clinical vignettes of serious medical conditions

All participants were presented with the same written clinical vignettes which described five acute medical conditions requiring more or less invasive treatments to prevent a fatal outcome. The five medical conditions were: 1, "suffering from pneumonia; antibiotic pharmaceutical treatment is necessary to save your life"; 2, "aneurysm that is likely to burst; only a surgical procedure can save your life"; 3, "paralysis of swallowing with risk of undernourishment and pneumonia due to inhaling food; a possible solution would be a percutaneous endoscopic gastrostomy surgical procedure for placing a feeding tube" (PEG); 4, "cancer is diagnosed; only chemotherapy may lead to life extension of one year"; 5, "comatose state after stroke; extension of life requires continuous artificial nutrition". Apparently scenarios 1 and 2 describe highly efficient treatments which can lead to a full recovery, while condition 5 defines the other extreme, and conditions 3 and 4 are in between.

Participants were asked to rate how likely they would decide today, ie, prospectively, to receive a life-maintaining treatment as described in each of the scenarios. Rating was performed on a five point Likert-scale with the following response categories: 0, "No, I certainly would not want that"; 1, "I see this rather critically"; 2, "I am still undecided on this matter"; 3, "Basically, I tend more to a decision in favour of treatment"; 4, "Yes, I certainly would want to receive treatment".

### Presentation of videos on living with dementia in the DCV group

Two short films (duration 6.5 minutes) were generated using scenes from the same documentary about an elderly demented woman. However, both films were tailored to give two different views on late-stage dementia. One film provided a selection of scenes in line with a deficit-oriented view of dementia, showing daily life situations which the demented person could not manage any more by herself ("negative film"). A voice-over with background commentaries by family members stressed the loss of previous abilities. Participants assigned to this film are referred to as "DCV-negative" (DCV-) group in the following.

Another film focused on the remaining resources of the demented woman. Therefore she was shown performing tasks she still was capable of ("positive film"). The aim was to present day-to-day tasks the way they are experienced by the affected person. That is without presuppositions. The persons remaining capabilities are not assessed with regard to her situation earlier in life. Still the demented condition of the presented person was clearly recognisable. Participants assigned to this film constituted the “DCV-positive” (DCV+) group.

### Consent to life-extending measures after watching the film

Subsequent to viewing alternatively one of the two video films a structured interview was administered in both DCV groups. The participants were asked again to respond to the same five medical scenarios as described above and were asked to rate again how likely they would prospectively decide for themselves to receive or refuse life-saving treatment if they were to live under similar circumstances as presented in the video just seen before.

### Assessment procedures for other variables

After the baseline assessment, participants answered questions about their education and completed a brief vocabulary test to assess crystalline intelligence (MWT-B) [28]. These measures serve to characterise and compare the three groups. Correlational analyses are outside the scope of the present paper.

### Statistical hypotheses and analyses

#### Main analysis

All analyses were conducted with SPSS-22. Effect sizes were computed using the R package “compute.es”. We specified and statistically tested two hypotheses: Firstly (hypothesis 1), we tested whether imagining oneself as being either demented or physically frail in the future would affect anticipated decisions regarding life-sustaining measures across a range of medical emergency situations. We tested this by comparing the average preference for a life-saving treatment versus no such treatment between the DCV and CCV groups at baseline, using an average (total) preference score across all five medical scenarios (see above). As subjects were randomly assigned to one of the groups, we conducted an independent sample t-test.

Secondly (hypothesis 2), we tested within the DCV group whether the presentation of a short film transmitting a positive image of living with dementia could modify such decisions, in contrast to a short film transmitting a negative image of dementia. In statistical terms, we tested whether the DCV+ group would exhibit higher post-film acceptance ratings compared to those in the DCV- group. Again we used the total score across all medical scenarios to test this hypothesis. As we randomly assigned members of the DCV group to one of the alternative films we conducted an ANCOVA on the post-film average acceptance scores as the dependent variable, with group membership (DCV+; DCV-) as a factor between subjects and the pre-film score as a covariate. We chose this approach over a repeated measures analysis as the response format was highly similar (same scenarios, same scale) but not identical (questionnaire at baseline versus interview after the film).

#### Exploratory analysis of scenario-specific effects

In addition, we explored whether acceptance rates regarding life-sustaining measures would differ between the five different scenarios. We expected such differences as the interventions vary considerably in their invasiveness and the associated impact on daily functioning. We therefore compared the pre-film rating scores of each scenario in the whole sample (DCV and CCV) using a repeated measurement ANOVA with Greenhouse-Geisser corrected degrees of freedom.

Furthermore, to explore whether the positive versus negative films would affect acceptance rates in the specific scenarios differentially, we repeated the main analyses for hypotheses 1 and 2 separately for each scenario. We tested for differences between DCV and CCV groups in pre-film acceptance rates for each scenario using independent samples t-tests (see hypothesis 1). Finally, we tested for differences in post-film scores for each scenario between DCV- and DCV+ group using a series of ANCOVAs (see hypothesis 2).

## Results

### Descriptive statistics of the sample

Table 1 gives the sample characteristics of the whole study sample and separately for the groups receiving either the Control Case Vignette (CCV) or the Dementia Case Vignette (DCV) with subsequent presentation of a film portraying either a positive or negative image of living a life with dementia (see Method section for further details). As expected from the randomised allocation, the two DCV groups and the CCV group did not differ statistically regarding age, education or vocabulary.

**Table 1:** Description of demographical data of the study sample (n = 278).

	Whole sample (n = 278)		DCV, positive care situation (n = 88)		DCV, negative care situation (n = 97)		CCV (n = 93)	
	Mean	(SD)	Mean	(SD)	Mean	(SD)	Mean	(SD)
Age (years)	53.36	(17.52)	54.16	(16.51)	54.51	(18.36)	51.40	(17.56)
Education (school years)	11.91	(1.37)	11.85	(1.40)	11.85	(1.41)	12.01	(1.31)
MWT-B	31.18	(3.45)	31.61	(2.84)	30.82	(3.96)	31.14	(3.41)

DCV: Dementia Case Vignette; CCV: Control Case Vignette; MWT-B: Vocabulary intelligence test [28]

### Main analysis

**Fig 2:**
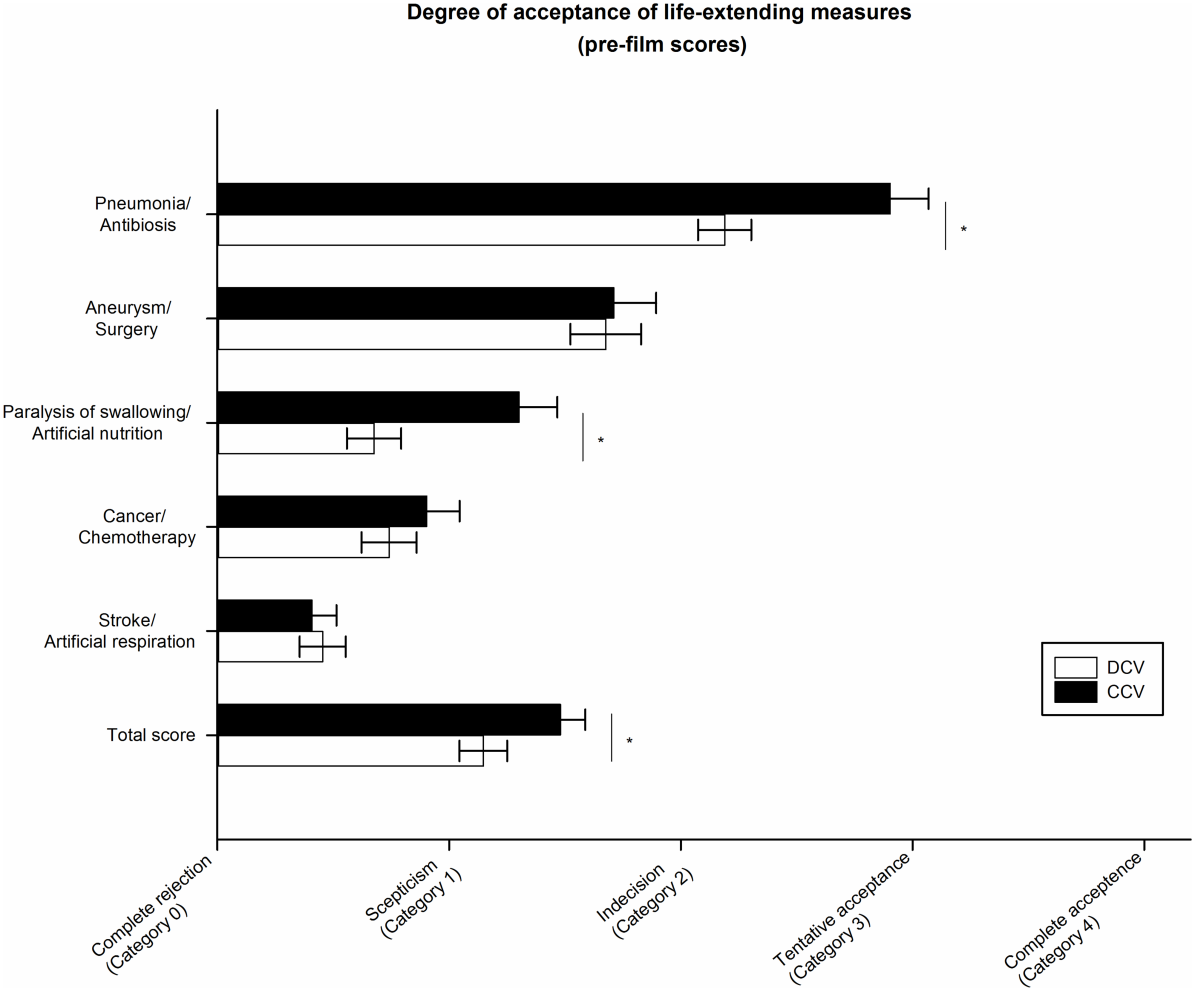
Difference in the acceptance of life- extending measures overall and according to each scenario at baseline. Labels on the X-Axis are paraphrases of the original Likert-Scale response categories. DCV: Dementia Case Vignette, CCV: Control Case Vignette. * Significant difference between groups (p<0.05).

In step 1 (testing hypothesis 1) we examined whether the average preference for life-sustaining measures would depend on the imagined health context of either being old, frail but competent, or demented and decisionally incompetent. In line with hypothesis 1, the DCV group was significantly less inclined to accept life-extending measures than the CCV group (t = 2.645, df = 276, p = 0.009, Cohen’s d = 0.34 (CI: 0.08–0.59)). Fig 2 illustrates the group differences in the ratings of acceptance of life-extending measures before film intervention between individuals assigned to the Dementia Case Vignette (DCV) and those assigned to the Control Case Vignette (CCV). Displayed are mean pre-film scores separately for each medical scenario (see exploratory analysis below for statistical results) and for the total score, averaged across all five medical scenarios.

Step 2 (testing hypothesis 2) addressed whether attitudes towards life-sustaining measures in the DCV group can be modified by short positive or negative films on living with dementia.

**Fig 3:**
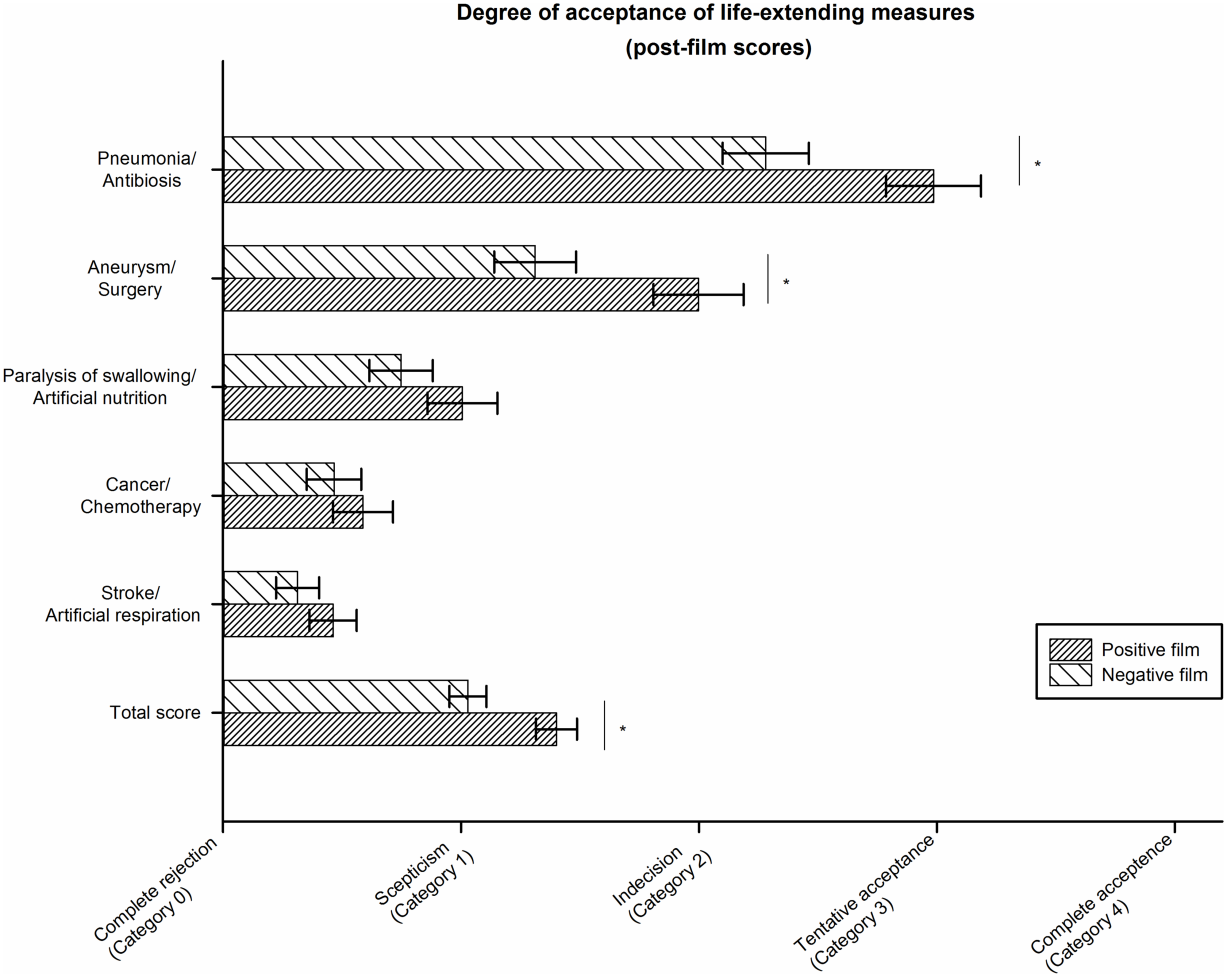
Difference in the acceptance of life-extending measures in the Dementia Case Vignette (DCV) groups after presentation of two alternative films conveying either a positive or negative image of living with dementia. Labels on the X-Axis are paraphrases of the original Likert-Scale response categories. DCV: Dementia Case Vignette, CCV: Control Case Vignette. * Significant difference between groups (p<0.05).

Fig 3 shows the post-film global acceptance ratings for individuals in the pre-film DCV group who were afterwards randomly assigned to watch a film portraying a positive image of a life with dementia (DCV-positive film) versus those individuals of the DCV group randomly assigned to a negative image film (DCV-negative film). Displayed are post-film ratings separately for each medical scenario (see exploratotry analysis for statistical results) and for a total score, i.e. averaged across all five medical scenarios. Values are adjusted for pre-film scores by ANCOVA.

The analysis of the averaged post-film preferences revealed a significant main effect (F(1,181) = 7.05, p = 0.009, partial Eta^2^ = 0.04), such that the DCV+ group had higher post-film global acceptance scores than the DCV- group (Cohen’s d = 0.45 (CI: 0.16–0.74)). Thus, our second hypothesis that the presentation of a short film conveying a positive image of a life with dementia would have a positive impact on subsequent global acceptance ratings, was also supported by the data.

### Exploratory analysis of scenario-specific effects

The explorative analysis of the scenario-specific pre-film acceptance rates revealed significant differences between the scenarios (F(3.354,929.066) = 176.251, p<0.001, Eta^2^ = 0.39). Post-hoc t-tests showed significant differences between all scenarios (t(277)>5.34, all p<0.001) with the exception of scenario 3 (paralysis of swallowing) and scenario 4 (cancer) (t(277) = 1.14, p = 0.256). Life-extending measures were most often endorsed for the acute conditions 1 (pneumonia) and 2 (aneurysm), where efficient treatments usually lead to a full recovery.

With regard to hypothesis 1, comparison of pre-film acceptance rates between the DCV and CCV groups demonstrated that acceptance of life-extension in the DCV group was significantly lower than in the CCV group only in scenario 1 (antibiosis; t(276) = -3.57, p<0.001, d = 0.45 (CI: 0.20–0.71)) and scenario 3 (paralysis of swallowing; t(137.4) = -3.4, p = 0.001, d = 0.49 (CI: 0.23–0.74), see Fig 2.

With regard to hypothesis 2, the scenario-specific analyses revealed that significant effects of the positive film intervention on post-film acceptance rates were confined to scenario 1 (antibiosis; F(1,181) = 4.85,p = 0.029, Eta^2^ = 0.03) and 2 (aneurysm; F(1,181) = 4.61,p = 0.033, Eta^2^ = 0.07) (Cohen’s d = 0.54 (CI: 0.24–0.83) and d = 0.48 (CI: 0.18–0.77), respectively; ie, medium effects sizes, see Fig 3). Importantly, these scenarios also had the highest pre-film acceptance rates and both represent situations in which full physical restitution after intervention is expectable without a strong negative impact of daily functioning.

## Discussion

Advance directives require the projective imagination and judgement of a range of possible future impairments, disabilities and medical situations for oneself. When deciding on their own treatment preferences in future life-threatening conditions participants in this study were very restrained towards life-saving interventions in end-of-life scenarios (either being demented or frail). Only antibiotic treatment of pneumonia in the control condition was on average tentatively accepted.

This study has two major findings relevant for understanding and supporting this complex decision-making process which is the basis for setting up advance directives:

First, imagining oneself as being demented, in contrast to imagining oneself as being frail but mentally competent, has a negative impact on the overall acceptance of life-extending measures. Detailed analysis of the five scenarios revealed that those significant differences only occurred under less life-threatening conditions: particularly in scenario 1 (pneumonia/antibiosis) and scenario 3 (paralysis of swallowing/artificial nutrition).

The second major finding is that the general dementia-related negative bias against certain life-supporting measures can be modified by viewing a short film conveying a positive image of living with dementia. This effect was found only, with medium effect sizes, for efficient, immediately life-saving interventions (antibiosis for pneumonia, surgery for aneurysm). For both of these scenarios the mean pre-film ratings indicated that participants were "undecided on this matter". In contrast, the other scenarios had mean pre-film ratings in the range of skepticism down to complete rejection. These pre-established declining scenario-specific attitudes towards life-sustaining measures may have precluded a modification via the film-intervention.

Taken together, the pattern of findings (pre- and post-film attitudes) suggests that a deficit-oriented view on dementia substantially contributes to the reservation towards life-extending measures for imagined medical situations; for situations which can be treated effectively. The demonstration that these preconceptions can be modified by providing visual information support the suppositions that "erroneous anticipations" of preferences in a life-threatening scenario are possible [17]. Conveying a positive image of living with dementia increases the willingness to consider life-saving measures for oneself in the imagined state of being demented.

It is plausible to assume that this life-affirming effect can be strengthened by more detailed and emotionally appealing information on dementia in an easily comprehensible and accessible manner. It is very likely that preconceptions about dementia are formed by an overbearing anticipation of losing capabilities. Yet, a person in a dementia state might not be aware of the loss of former abilities but might just live within the present, without reference to the past and the future. A positive perspective on dementia can significantly increase the sensibility and awareness of how such illness patterns can affect not only others but one’s own personal health. Subjects currently considering advance directives may make better informed decisions based on empathic and compassionate experiences and attitudes when they have access to media which also include the positive sides of a living with dementia. In this context it is recommended that clinicians strengthen the empathic perspective on dementia and tell patients about the empirical evidence that patients with dementia can adjust to their disabilities [29]. By these means, acceptance of a life with dementia might be increased.

The present study is in line with the study by Volandes et al. [30] in showing that video clips of living with dementia can impact on treatment preferences. In the study by Volandes et al. [30] overall preferences regarding the goals of advanced care were examined. After watching a video showing a demented woman unable to communicate, ambulate or eat on her own, participants more often preferred "comfort care" over any invasive, "live-prolonging" care. The present study extends the findings of Volandes et al. [30]: viewing a more positive video on living with dementia, which is highlighting resources and not only handicaps and impairments, is able to shift preferences towards life-saving interventions, at least under specific medical circumstances. The statistically significant effects were of medium effect size, and may not be considered to be “clinically relevant”. We also do not know whether the effects would be lasting. However, our goal was not to evaluate a specific and ready to use tool for better advance directives. Rather we aimed to show that treatment preferences relevant for advance directives are modifiable. The positive findings obtained may inform the future development of media tools to support decisions in advance directives.

### Strengths and limitations

*Strengths*: This study is among the first to examine the effects of general preconceptions about living with dementia on advance directives, and proved the short-term modifiability of such effects by audiovisual information. For several specific medical situations hypothetical decisions were to be taken. The selection of life-threatening medical situations comprises the usual scope of medical decisions represented in official recommendations for advance care planning. The scenarios differed by the associated benefit/risk ratios and prospective life expectancy risks.

*Limitations*: Although questionnaire responses were used at baseline and an interview format was used after the film, the assessment of acceptance of life-extending measures in the DCV group was based on the same medical scenarios, and participants will have remembered their previous responses. Research in social psychology has shown that subjects seek consistency in behaviour in social situations to maintain their credibility [31]. In fact we observed a strong and highly significant covariate effect of the baseline score on post-film scores with a partial Eta^2^ of 0.69. These memory and consistency effects may have led to a restricted and possibly underestimated effect of our intervention. Furthermore, we cannot exclude that perceived illness severity was systematically different between the two different videos, despite the fact that both videos were created from the same, longer video showing the daily life of one demented patient and his family. A perceived severity effect might either have added to, or conversely, could have reduced the effects of the indended manipulation regarding resources versus deficits in dementia. However, this does not challenge, but rather emphasizes our conclusion that the perception of a disease is affected by the framing and selection of information about the disease.

### Policy implications and future research

We demonstrate that projecting treatment preferences in future states of illness after losing cognitive capacities is dependent on visualized information by video clips regarding the possibility to experience quality of life despite of dementia. This observation has implications:

Advance directives should not be formulated in a too global fashion (eg, in case of incompetence and dementia: "life-saving measures should not be taken". Confronted with specific life-threatening scenarios and their different benefit/risk ratios, preferences of still competent subjects for medical interventions strongly depend on the specific scenario: effective interventions with a relatively low risk which promise to be life-saving are substantially more often preferred than high-risk interventions with a less likely positive outcome.

An implicit assumption of the instrument of "advance directives" is that the anticipation of one’s own treatment preferences under different medical conditions in the distant future can validly be achieved (precedent autonomy). Yet, the subjective attitudes in this respect are not always well-considered, they can be based on insufficient information and might be prejudiced particularly if social stereotypes are prevailing (as it is the case for dementia). Advance directives concerning dementia therefore should be based on scenarios that present the affected persons perspective in a realistic manner including the possibility of experiencing positive emotions also in this state.

As shown in this study, these attitudes of cognitively fully competent subjects towards living with dementia can be shifted by visual information (videos films) toward life affirmation. Extrapolating these findings to the societal level, we suggest that awareness campaigns could modify the stereotypic, negative view of dementia.

The prospective validity of advance directives presumes the stability of treatment preferences (as long as a competent person does not revoke the advance directive). Given the uncertainties of the medical progress and of living with severe dementia, beliefs about the quality of life in this stage might change over time. Thus, it is required to prevent the impending conflicts between previous anticipations and future real experiences. Regular reconsideration of previously formulated advance directives—oriented at the actually available empirical evidence—should therefore be systematically implemented in order to prevent erroneous anticipations.

The current study proposes that video support tools could be useful for medical counselling for advance directives in case of dementia. Furthermore, public awareness and anti-stigma campaigns for dementia will support competent adults in formulating their critical interests for the case to experience dementia later in life less biased by the negative deficit-oriented image of this disease.

## Supporting information

S1 Study data Overview. (XLSX)
